# 

**Published:** 1884-01

**Authors:** 


### INDEX TO VOLUME XXXVIII.

A Good Antiseptic.................. 52
A New Department................. 55
A New Clamp......................... 56
Antiquity of the Trephine...... 101
A Simple Method of Producing a Reticulated Surface, on the Palatal Side, of Dental Base-Plates...................... 139
Acid and Fungus.................... 139
A New Combination.............. 160
A Proposition........................ 212
Address to the Graduates......... 224
A New Observation in Dentinal Structure........................ 245
Antram, Engorgement of......... 608
Adapting Artificial Crowns to Roots of Teeth................ 280
A Supernumary Tooth Above the Superior Third Molar.. 305
A Swarm of Quacks; Medical College; Old Times........... 351
American Dental Association... 365
Antiseptics and Disinfectants... 402
A Bar to Malpractice Suits...... 395
A Resume of Treatment of Alveolar Abscesses.............. 421
A Plea for Education.............. 431
An Old Vulcanite Plate.......... 433
A Bogus Directory................. 434
Association of State Boards of Dental Examiners........... 446
American Dental Association... 472
A Test for the Purity of Iodoform ............................ 563
A Case................................ 579
Biographical.......................... 7
Book Notices......................... 109
Book Notices........................ 161
Beer Drinking in Relation to Health and Longevity........ 290
Book Notice.......................... 314
Board of Examiners............... 526
Borax and Iodide of Potasah for the Voice.................... 565
Bibliographical................. 577–622
Correspondence...................... 45
Commencement .............. 108–621
College Associations............... 109
Corrosive Sublimate as an Antiseptic............................ 142
Correspondence..................404, 154
Carbolic Acid....................... 173
Cause and Treatment of Facial Neuralgia....................... 243
Care of Childrens' Teeth.......... 321
Comparative Dental Anatomy.. 328
Cause of Spasm of Muscles of Face, and Cataract, Due to Dental Irritation............. 389
Chloride of Sodium in Inflamatory Diseases.................. 463
Chemistry ............................ 479
Chloroform Syncope Treated by Reversing........................ 559
Cinnamon Bark for Tooth-ache.. 565
Dental Society State of New York ............................ 214
Dentists' Benevolent Association ............................. 366
Delegates to the American Dental Association................ 367
Dental Education.................. 369
Dental College Association...... 471
Drowned ............................ 472
Dentists' Benevolent Associat'n 527
Dentistry in Austria.............. 529
Dental Caries.................. 564–623
Extracts ............................. 248
Errors in Diagnosis................ 256
Educational ......................... 262

INDEX.

Editorial ... 266
Editorial ... 311
Editorial ... 365
Editorial ... 418
Extension of Term ... 418
Electrical Surgery ... 464
Editorial ... 618, 468
Experiments in Modelling Composition ... 552
Food, Animal and Vegetable ... 598
Fortieth Annual Meeting of the Mississippi Valley Dental Society ... 184
First Aid to the Injured ... 290
Feeding Infants ... 395
Horse-Back Riding and Pulmonary Calisthenics ... 143
How to Keep the Hypodermic Syringe in Order ... 255
Hydrophobia—Prevention and Cure ... 307
How to Secure Good Dental Organs; Prevent Their Decay; Prevent Rickets, Hip Disease, etc ... 326
Hysterics and Neurasthenia Caused by Dental Irritation ... 532
How to Prepare a 1 to 1,000 Solution of Corrosive Sublimate ... 566
Iodoform Intoxication ... 146
In Memoriam ... 313
Important Decision ... 316
Improvement in Artifical Dentures ... 581
Irregularity—A Practical Case.. 317
Injurious Effects of a Rubber Plate ... 355
Indiana Dental College ... 419
Iodoform in Dental Surgery ... 449
Letter from London ... 407
Letter from Geneva ... 409
Lost—Registers ... 420
Local Anesthesia ... 537
Lemon Juice in Malaria ... 562
Law ... 573
Miscellany ... 40
Mississippi Valley Dental Society ... 106
Micro Organisms in Water ... 148
Malaria Night Blindness ... 148
Michigan Dental Society ... 161
Mad River Dental Society ... 214
Meeting ... 316
Meeting of Delegates ... 357
Minnesota State Dental Society.. 367
My Faliures ... 382
Minnesota State Dental Society.. 418
Married ... 420
Meeting of College Faculties... 436
Mad River Valley Dental Society ... 539
Minnesota State Dental Society.. 544
Minnesota Medical Practice Act. 557
Medical Education ... 564
Membrane of an Egg for Skin Grafting ... 566
New Remedies ... 277
Neuralgia Pencils ... 291
National Association, No. 2 ... 303
Nerve as a Barometer ... 350
National Association of Dental Examiners ... 366
Neuralgia Caused by Exostosis.. 560
Northwestern Dental Association ... 579
Ohio State Dental Society—Reorganized ... 618
On Erosion ... 18
On the Treatment of Pulpless Teeth ... 88
Old Shoes ... 101
On Certain Microscopic Elements in Pulpless and Gum-Denuded Teeth, in Their Relation to the Filling of Roots, and the Reattachment of the Gum Tissue.... 111
Odonto-Periostal Tumor Simulating Pulp-Fungoid ... 152
Operative Dentistry ... 232
On Proposed National Dental Association ... 356
Odontological Society of Great Britain ... 461
Obituary ... 528
On the Removal of Naso-Pharyngeal Tumors ... 534
On the Action of a Secretion Obtained from the Medical Leech ... 558
Obituary ... 579–626
Oil of Cloves to Remove the Antipathy Against Chloroform ... 611
Prosthetic Dentistry ... 13
Physician's Visiting List for '84 56

Personal.... 56
Pyorrhea Alveolaris the Result of Disease.... 59
Phosphate of Lime and the Teeth 163
Proceedings of Societies.... 252
Patients will Lie.... 255
Professional Duties and Practical Suggestions.... 267
Professional Attainments and Popular Needs.... 274
Proceedings of the Northern Ohio Dental Association.... 338
Pennsylvania State Dental Society.... 366
Pennsylvania State Society.... 368
Proper Medical Education.... 468
Proceedings and Discussion of the Twenty-fourth Annual Meeting of the American Dental Association.... 486
Removal of the Greater Portion of Both Upper Jaw-bones without External Incision.. 39
Resolutions on the Death of Dr. A. L. Talbert.... 57
Report on the Teeth of a Mound Builder.... 82
Reflex Nervous Influence.... 95
Replantation and Transplantation of Teeth.... 98
Rules of Business.... 136
Removal of the Outer Plate of the Skull Following a Severe Burn.... 284
Rendering Rubber Tube Odorless.... 289
Robinson's Fibrous and Textile Filling.... 302
Removal of Warts.... 396
Reflex Pain.... 473
Sugar as a Dressing for Wounds 42
Salmagundi.... 102
Salmagundi.... 154
Salmagundi.... 208
Scientific American.... 110
Storage Battery Light.... 142
Salmagundi.... 257
Salmagundi.... 308
Salmagundi.... 612, 358
Stammering—Its Relation to Poverty and its Treatment. 287
Selling His Practice.... 289
Sarcoma of the Superior Maxilla in a Child of Eight.-Removal 291
Synopsis.... 293
Synopsis of the Annual Meeting of the Illinois State Dental Society.... 329
Some of the Dangers and Disadvantages of Anæsthesia.. 341
Supreme Court of Illinois.—Opinion.... 352
Society Meetings.... 367
Small Doses Frequently Repeated.... 393
Salmagundi.... 411
Salmagundi.... 465
Salmagundi.... 522
Scientific Fallacies.... 561
Salmagundi.... 570
The Value of Definite Therpeutical Combination.... 37
The Lack of Interest in Hygiene 40
The Common Diseases of Children.... 44
Temperance and Longevity.... 99
The Immediate Suture of Divided Nerves.... 101
To be Continued.... 108
The Relation of Physiological Study to Pathology and Therapeutics.... 140
The Folly of Anti-Vaccinators.. 144
Trees in Towns.... 147
The Causes and Prevention of Suicide.... 148
The Action of Saline Cathartics 150
The Use of Tobacco by Boys.... 150
The Alabama State Dental Association.... 160
The Southern Dental Association 213
The Debts We Owe.... 215
The M. D.'s.... 256
Two Cases of Death after Extraction of Teeth.... 292
The Nutritive Functions.... 585
The Causes of Failure in Dental Operations.... 377
The Promotion of Osseous Development.... 397
The Hippocratic Oath.... 392
The Hypodermic Injection.... 395
Tongaline.... 396
The Writing of Galen.... 396
To Saratoga.... 419
To Disguise the Taste of Iodide of Potash.... 448
The Sanitary Condition of To-day 464

INDEX.

Transactions of the Annual Meeting of the Illinois State Dental Society......... 472
The Ohio State Dental Society... 525
The Robinson Remedy............ 550
The Men Who are Promoted.... 562
To Cure Stammering............... 569
The Value of Vaccination........ 569
The New Journal of Dentistry.. 575
The Ninth Meeting of the International Congress............. 576
What is Meant by Nervous Prostration ....................... 126
White Fillings......................... 143
What to do with our Sons........ 147
Why is Chromic Acid such a Valuable Caustic?............ 255
What Chauncy Depew Says...... 460
Very Successful...................... 352
Vicks Floral Guide................. 53

### DIRECTORY OF DENTAL SOCIETIES.

*American Dental Association*—Meets on the 1st Tuesday of August, 1884, at Saratoga. Pres. E. T. Darby, Philadelphia, Pa.; Cor. Sec. A. W. Harlan, Chicago, Ill.; Rec. Sec. Geo. H. Cushing, Chicago, Ill.

*National Dental Association*—Meets Thursday, in Washington City, Aug. 3, 1884. Pres. Jno. B. Rich, New York; Sec. R. Finley Hunt, Washington, D. C.

### STATE DENTAL SOCIETIES.

*Alabama Dental Association*—Meets annually. Next meeting at Birmingham, on the second Tuesday of April, 1884. Pres. E. S. Chisolm; Sec., E. Wagner.

*California State Dental Association*—Tuesday, May 22d, 1884. Pres. Wm. Dutch, San Francisco; Sec. S. E. Goe., San Francisco; Cor. Sec. H. E. Knox, San Francisco.

*Connecticut State Dental Society*—Pres. R. W. Brown, New London; Rec. Sec. Geo. L. Parmelee, Hartford. Annual meeting third Tuesday in May, 1884, at Hartford.

*Dental Society of the State of Maryland and District of Columbia*—Meets annually. Next meeting at Washington City, on the second Tuesday in October, 1883. Pres. B. F. Coy, of Baltimore: Sec. M. W. Foster, M. D.

*Georgia State Dental Society*—Meets second Monday in May, 1884, at Atlanta, Ga. Pres Geo. H. Winkler Augusta; Rec. Sec. G. W. H. Whitaker, Sandersville; Cor. Sec. L. D. Carpenter, Atlanta.

*Illinois State Dental Society*—Meets annually. Pres. Ewd. Noyes, Chicago; Sec G. W. Wassal, Chicago. Next place of meeting, Decatur, second Tuesday in May, 1884.

*Indiana State Dental Society*—Meets next year at Indianapolis, on the last Tuesday of June, 1883. Pres. J. E. Cravens, Indianapolis; Sec. Robert Van Valzah, Terre Haute.

*Iowa State Dental Society*—Meets annually. Next meeting in Council Bluffs, on the first Tuesday in May, 1884. Pres. E. E. Hughes, Des Moines; Rec. Sec. J. B. Montfort, Fairfield.

*Kentucky State Dental Association*—Meets annually, first Tuesday in June, 1883. Next meeting in Louisville. Pres. Dr. Hooper, Owenton; Sec. E. C. Dunn, Louisville.

*Kansas State Dental Association*—Pres. A. N. Callahan, Topeka; Sec. J. D. Patterson, Lawrence. Next meeting will be held at Hiawatha, Kansas, in joint session with the Nebraska State Society, first Tuesday in May, 1884.

*Maine Dental Society*—Meets in August, at Thomaston. Pres. E. J. Robert, Augusta, Sec. C. E. Hussey, Beddeford.

*Maryland State Dental Association*—Meets annually in October. Pres. E. J. Smithers; Cor. Sec. Wm. A. Mills; Rec. Sec. B. M. Hopkinson.

*Massachusetts Dental Society*—Meets annually in Boston, second Thursday in December. Pres. F. Searl, Springfield; Rec. and Cor. Sec. W. E. Page, Boston.

*Michigan State Dental Association*—Meets annually. Next meeting at Detroit, last Wednesday in March, 1884. Pres. W. H. Dorrance, Ann Arbor; Sec. J. B. McGregor; Treas. J. Lathrop, Detroit.

*Mississippi State Dental Association*—Will meet third Tuesday of June, 1884, at Jackson. Pres. Geo. W. Rembert; Sec. R.G. Miller, Jackson.

*Minnesota Dental Association*—Meets annually. Meets May 26, 1884, at Winona. Pres. W. F. Lewis, Sec C. M. Bailey, Minneapolis.

*Missouri Dental Association*—Meets annually on the first Tuesday of June, 1884, Next meeting at Sweet Springs. Pres. A. N. Fuller; Sec. W. H. Eames, St. Louis.

*Nebraska State Dental Society*—Meets annually. Next meeting first Tuesday in May 1884, at Hiawatha, Kansas, in joint session with the Kansas State Dental Society. Pres. J. W. Funck, Beatrice; Sec. W. F. Roseman, Fremont.

*New Jersey State Dental Society*—Meets annually. Next meeting at Long Branch. on Wednesday, July, 20th 1883. Pres. F. A. Levey, Orange; Sec. C. A. Meeker, Newark.

*New Hampshire Dental Society*—Pres. D. W. Eagerly, Farmington; Sec. C. W. Clerment, Manchester.

*North Carolina State Dental Society*—Meets annually. Next meeting at Raleigh, second Tuesday in June, 1884. Pres. W. H. Hoffman; Sec. Thos. M. Hunter, Fayetteville.

*Ohio State Dental Society*—Meets annually at Columbus, last Wednesday of October, 1884. Pres. A. Berry, Cincinnati; Sec. J. H. Warner, Columbus.

*Oregon State Dental Society*—Meets annually. Pres. J. R. Cardwell, Portland; Rec. Sec. S. J. Barber.

*Pennsylvania State Dental Society*—Meets Tuesday, July 27th, 1884, at Wilkesbarre; Pres. S. H. Guilford, Philadelphia; Rec. Sec. E. P. Kremer, Lebanon, Pa.; Cor. Sec. W. H. Fundenberg, Pittsburgh.

*Rhode Island Dental Association*—Pres. A. W. Buckland, Woonsocket; Sec. L. L. Buckland, Providence.

*South Carolina State Dental Association*—Meets annually. Pres. B. A. Muckenfuss; Sec. G. F. S. Wright, Columbia; Cor. Sec. R. A. Smith. Next meeting at Cheraw, May 3, 1884.

*Texas State Dental Association*—Meets in the city of Austin, May 4, 1884. Pres.—— Sec. W. R. Clifton

*Tennessee Dental Association*—Meets Annually. Next meeting at Nashville third Tuesday in February, 1884. Pres. J. H. Prewitt, Madisonville, Ky.; Rec. Sec. H. W. Morgan, Nashville; Cor. Sec. J. F. Crawford, Nashville.

*The Dental Society of the State of New York*—Meets annually on the second Wednesday in May. Next session at Albany, May 12, 1884. Pres. L. S. Straw, Newburgh; Rec. Sec. S. A. Freeman, Buffalo; Cor. Sec. W. H. Atkinson, New York.

*Vermont State Dental Society*—Meets annually. Next meeting at Burlington, March 15, 1884. Pres. L. T. Lawton, Rutland, Sec. C. F. Lewis, Burlington.

*Virginia State Dental Association*—Meets in Charlottsville, Tuesday, August 19, 1883. Pres. J. Hall Moore; Sec. L. M. Cowarden, Richmond.

*Wisconsin State Dental Society*—Meets annually. Next meeting at Milwaukee, July 20, 1884. Pres. J. S. Reynolds, Monroe; Sec. R. G. Richter, Milwaukee.

### LOCAL SOCIETIES

*American Academy of Dental Science*—Meets annually in Boston, Mass., on the last Wednesday in October, 1883. Pres. J. L. Williams; Rec. Sec. Jno. T. Codman; Cor. Sec. C. P. Wilson, Boston.

*Brooklyn Dental Society*—Meets monthly—second Monday of each month. Pres. A. H. Brockway; Rec. Sec. A. P. Crandall, 508 Clinton Ave.

*Central Dental Association of Northern New Jersey*—Meets annually in February. Pres. C. A. Meeker, Newark, Sec. G. Carleton Brown, Elizabeth.

*Central Illinois Dental Society*—Meets annually. Next meeting at El Paso, Oct. 9, 1883; Pres. ——; Sec. C. R. Taylor, Streetor.

*Chicago Dental Society*—Prest., E. S. Talbot; Sec. H. F. Kimball; Cor. Sec. A. W. Harlan.

*Cincinnati Dental Society*—Meets on the second Tuesday in each month. Pres. W. D. Kempton; Sec. A. G. Rose.

*Connecticut Valley Dental Society*—Meets 27 and 28 of Oct. 1884, at Savin Rock Conn. Pres. C. T. Stockwell, Springfield, Mass. Sec. A. M. Ross, Chicopee.

*Eighth District Dental Society, N. Y.*—Meets semi-annually. Next meetlng in Buffalo, April 15, 1884. Pres. S. A. Freeman; Sec. C. S. Butler, Buffalo.

*East Tennessee Dental Association*—Meets annually. Next meeting will be held at Chattanooga, Tenn., on the first Tuesday of Aug., 1884. Pres. R. A. B. Noyers, Sec. S. N. Prothro.

*New Hampshire State Dental Society*—Meets annually. Pres. H. Hill, Manchester; Sec. E. B. Davis, Concord.

*Merrimac Valley Dental Association*—Meets annually. Pres. J. N. Chandler, Boston; Sec. H. W. Coburn, Lowell, Mass.

*Mississippi Valley Dental Society*—Meets annually at Cincinnati, on first Wednesday in March, 1884. Prest. Prof. C. M. Wright, Cincinnati; Sec. W. H. Cameron, Cincinnati.

*New England Dental Society*—Meets annually.

*Nashville Dental Association*—Meets on the second Tuesday of each month. Pres. R. R. Freeman; Sec. L. G. Noel, Nashville.

*Northern Ohio Dental Association*—Meets annually. Next meeting at Sandusky, O., on the second Tuesday in May, 1884. Pres. D. Gale French of Pittsburg, Pa.; Rec. Sec. A. J. Douds, Canton; Cor. Sec. H. F. Harvy, Cleveland, O.

*Northwestern Dental Association* (embraces Dakota, Minnesota and Manitoba) meets annually. Next meeting at Grand Forks, Dakota, fourth Tuesday in July, 1884. Pres. Lewis Ottofy, Grand Forks, Dak.; Sec. S. J. Hill, Fargo, Dak.

*Ohio Dental College Association*—Meets annually, Tuesday, March 2, 1884, at Cincinnati. Pres. H. M. Reid, Cincinnati; Rec. Sec. F. A. Hunter, Cincinnati.

*Odontographic Society of Pennsylvania*—Meets on the first Wednesday of each month at 8 o'clock p. m., in the Philadelphia Dental College. Pres. J. L. Eisenbrey. Rec. Sec. E. L. Hewitt.

*Odontological Society of New York City*—Meets the third Tuesday of each month. Pres. S. G. Perry; Rec. Sec. E. T. Payne.

*Pennsylvania Association of Dental Surgeons*—Pres. J. H. Githens. Rec. Sec. Theo. F. Chupein.

*Pittsburg Dental Society*—Meets the first Tuesday Evening of each month. Pres. F. A. Reinhart; Sec. W. N. Fundenburgh.

*Fifth District Dental Society of N. Y.*—Meets semi-annually. Next meeting in Syracuse, on the 12th of October, next. Pres. E. L. Swartwout, of Utica. Sec. I. C. Curtis of Fulton.

*San Francisco Dental Society*—Meets monthly. Pres. Alex. Warner: Sec. A. W. Knowles; Treas. J. J. Birge.

*Sixth District Dental Society of N. Y.*—Meets semi-annually. Next meeting at Cortland, Thursday, Oct. 6th, 1883. Pres. L. E. Freeland, Onconta; Sec. A. J. Wright, Oswego.

*Washington Dental Society*—Meets second Tuesday of each month. Pres. R. Finley Hunt; Rec. Sec. H. M. Schooly.

*Southern Dental Association*—Meets in Lexington, Ky., on the first Tuesday in May, 1884. Pres. H. J. McKellops, St. Louis, Mo.; Cor. Sec. J. P. Holmes, Macon, Ga.; Sec. W. H. Hoffman.

*First District Dental Society of the State of New York*—Meets annually. Pres. A. L. Northrup; Sec. J. E. Dexter, New York.

### EXCHANGES.

St. Louis, Medical and Surgical Journal.
The Pacific Medical and Surgical Journal, San Francisco.
Cincinnati Lancet and Clinic.
Dental Cosmos, Philadelphia.
Chicago Medical Examiner.
The Detroit Review of Medicine and Pharmacy.
Medical Record, N. Y.
American Journal of Dental Science, Baltimore.
Indiana Journal of Medicine.
Missouri Dental Journal, St. Louis.
The Sanitaran, New York City.
L'art Dentaire, Paris.
Le Progress Dentare, Paris.
Deutische Monatsschrift, Leipzig.
Physician and Surgeon, Ann Arbor, Mich.
Ohio State Journal of Dental Science, Xenia, Ohio.
The Microscope, Ann Arbor, Mich.
Scientific American, New York.
Independent Practitioner, N. Y.
The Dental Record, London.
The Journal of the British Dental Association, London.
The Southern Dental Journal.
Items of Interest, Philadelphia.
The Dental Practitioner.—The Cincinnati "Medical" News.
The New England Journal of Dentistry.

### Personal.

All the readers of the Register have some knowledge of, and many of them well know Dr. Geo. W. Field, formerly of Cincinnati, who made an almost world-wide reputation as an American dentist in Geneva, Switzerland, and subsequently removed to London, and there established a lucrative practice, which taxed his health so severely that he was compelled to abandon it. He returned to the United States and devoted three or four years in recuperation. His health being fully restored he has, within the past few months, returned to London and resumed practice, in which his friends wish him abundant success. He is located at 23 Park street, Park Lane, London, W.

### A New Clamp.

I have used for several months past a rubber dam clamp, devised and constructed by Dr. J. P. Carmichael, of Milwaukee. It is a double clamp, the jaws of which consist of ten small steel plates, so hinged together as to make it adjustable, with a large

### MONTHLY LIST OF PATENTS

For Inventions relating to Dentistry and Surgery, bearing date December 25, 1883, reported for the DENTAL REGISTER, by Louis Bagger & Co.. Mechanical Experts and Solicitors of Patents, Washington, D. C.

No. 290,915. J. O'Brien, Kalamazoo, Mich., Artificial Legs.

No. 290,841. F. Bohsert, New York, N. Y., Surgical Chair.

No. 240,559. R. M. Ross and E. J. Klinck, Utica, N. Y., Dental Engine Fan.

No. 290,401. W. P. Cooke, Milford, Mass., abrasing disk-holding Spindle for Dental Engine.

No. 290,608. J. W. Snyder, Philadelphia, Pa., Medicinal Inhaler.

No. 10,423. R. M. Ross, Attica, N. Y., Dental Drill Hand Piece.

No. 290,814. B. F. Eshelmans, Harlan, Iowa, Dental Plugger.

No. 289,472. W. W. Vance, Kearney, Neb., Device for Holding Dental Drills on Whetstones.

No. 22,289,743. F. E. Young, Canton, Ohio, Surgical Chair.

### CONTENTS.

Biographical........ 7

COMMUNICATIONS.

Prosthetic Dentistry........ 13

On Erosion........ 18

SELECTIONS.

The Value of Definite Therapeutical Combination........ 37

Removal of the Greater Portion of Both Upper Jaw-Bones without External Incision........ 39

The Lack of Interest in Hygiene........ 40

Miscellany........ 40

A Protest Against Changes in Nomenclature........ 42

Sugar as a Dressing for Wounds........ 42

The Common Diseases of Children........ 44

Correspondence........ 45

Salmagundi........ 47

EDITORIAL.

A Good Antiseptic........ 52

Vick's Floral Guide........ 53

Suspended........ 55

A New Departure........ 55

Physicians' Visiting List for 1884........ 56

Personal........ 56

A New Clamp........ 56

Resolutions on the Death of Dr. A. L. Talbert........ 57

---

### THE DENTAL REGISTER,

A MONTHLY MAGAZINE DEVOTED TO THE INTERESTS OF THE

**DENTAL PROFESSION.**

*Terms Reduced to $2.00 Per Year. Single Copies, 25 Cents.*

**EDITED BY PROF. J. TAFT, D.D.S.**

—AND—

**Published by SPENCER & CROCKER, Ohio Dental and Surgical Depot.**

The volume commences in January and closes in December.

The REGISTER being issued monthly is always fresh, therefore Indispensable to the practitioner who desires to keep posted up with the new inventions and improvements which are daily developed. Neither expense nor effort will be spared to give value and variety to its contents. Articles will be illustrated with well executed engravings whenever they will add to the interest or assist to explanation.

Its extensive circulation and frequency of issue make it one of the BEST DENTAL ADVERTISING MEDIUMS in the United States.

All subscriptions must begin with January or July.

Local or special notices, five lines or less, will be inserted for $3 each insertion; over five lines, 50 cts. per line.

All advertising bills payable in advance, or at furthest, after the issue of the first number containing the advertisement. All transient advertisements to be paid for in advance.

☞CONTRIBUTIONS SOLICITED.

Exclusively Editorial Communications should be addressed to the Editor.

The latest number of the REGISTER may be ordered with or without other goods at any time, and all letters should be addressed to

**SPENCER & CROCKER, Ohio Dental & Surgical Depot, Cincinnati, O.**

